# Iterative Metal Artifact Reduction (iMAR) of the Non-adhesive Liquid Embolic Agent Onyx in Computed Tomography: An Experimental Study

**DOI:** 10.1007/s00062-021-01101-6

**Published:** 2021-10-13

**Authors:** Niclas Schmitt, Charlotte S. Weyland, Lena Wucherpfennig, Christian Herweh, Martin Bendszus, Markus A. Möhlenbruch, Dominik F. Vollherbst

**Affiliations:** 1 Department of Neuroradiology, Heidelberg University Hospital, Im Neuenheimer Feld 400, 69120 Heidelberg, Germany; 2 Department of Diagnostic and Interventional Radiology, Heidelberg University Hospital, Heidelberg, Germany

**Keywords:** Arteriovenous malformation, Embolization, Neuroimaging, Angiography, Vascular malformation

## Abstract

**Background:**

A drawback of Onyx, one of the most used embolic agents for endovascular embolization of intracranial arteriovenous malformations (AVM), is the generation of imaging artifacts (IA) in computed tomography (CT). Since these artifacts can represent an obstacle for the detection of periprocedural bleeding, this study investigated the effect of artifact reduction by an iterative metal artifact reduction (iMAR) software in CT in a brain phantom.

**Methods:**

Two different in vitro models with two-dimensional tube and three-dimensional AVM-like configuration were filled with Onyx 18. The models were inserted into a brain imaging phantom and images with (*n* = 5) and without (*n* = 10) an experimental hemorrhage adjacent were acquired. Afterwards, the iMAR algorithm was applied for artifact reduction. The IAs of the original and the post-processed images were graded quantitatively and qualitatively. Moreover, qualitative definition of the experimental hemorrhage was investigated.

**Results:**

Comparing the IAs of the original and the post-processed CT images, quantitative and qualitative analysis showed a lower degree of IAs in the post-processed images, i.e. quantitative analysis: 2D tube model: 23.92 ± 8.02 Hounsfield units (HU; no iMAR; mean ± standard deviation) vs. 5.93 ± 0.43 HU (with iMAR; *p* < 0.001); qualitative analysis: 3D AVM model: 4.93 ± 0.18 vs. 3.40 ± 0.48 (*p* < 0.001). Furthermore, definition of the experimental hemorrhage was better in the post-processed images of both in vitro models (2D tube model: *p* = 0.004; 3D AVM model: *p* = 0.002).

**Conclusion:**

The iMAR algorithm can significantly reduce the IAs evoked by Onyx 18 in CT. Applying iMAR could thus improve the accuracy of postprocedural CT imaging after embolization with Onyx in clinical practice.

## Introduction

Endovascular embolization of cerebral arteriovenous malformations (AVM) using liquid embolic agents (LEA) is an established treatment option [1–4]. The non-adhesive agent Onyx (Medtronic, Irvine, CA, USA), consisting of ethylene vinyl alcohol (EVOH) copolymer, dimethyl sulfoxide (DMSO) and radiopaque tantalum powder, is one of the most commonly used LEAs [5–7]. A well-known drawback of Onyx is the generation of imaging artifacts in computed tomography (CT) [8–12]. Since cerebral AVMs are associated with a risk of periprocedural and postprocedural hemorrhage, LEA-related imaging artifacts may represent a crucial obstacle for the detection of intracranial hemorrhage during or after embolization [13]. In addition, especially complex vascular malformations can often not be occluded completely by endovascular embolization alone and thus require subsequent surgical resection or radiation therapy [1]. The planning recordings for radiation treatment are usually based on CT imaging and LEA-related artifacts may represent a major obstacle for an appropriate and tissue-conserving treatment planning [14–18]. In the last few years, several acquisition and post-processing techniques for reduction of metal-related CT imaging artifacts have been introduced [19]. One of these is the iterative metal artifact reduction (iMAR) software (Siemens Healthineers, Erlangen, Germany). Several studies have shown its ability to reduce metal-related artifacts, especially for metal implants like intracranial coils or shoulder and hip protheses [20, 21]. The effect of iMAR on the artifacts evoked by Onyx in cerebral CT imaging has not been in the focus of research until now.

The aim of the present study was the investigation of the reduction of imaging artifacts caused by Onyx 18 using iMAR in conventional CT in two experimental in vitro models in a brain phantom.

## Material and Methods

### Preparation of the Experimental in Vitro Models

**Fig. 1:**
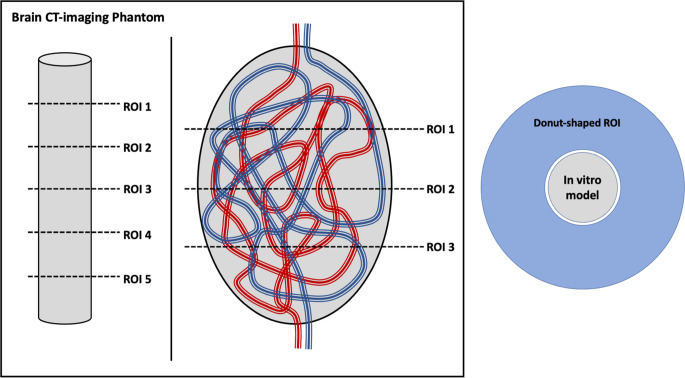
Schematic illustration of the experimental in vitro 2D tube and 3D AVM models. For the 2D tube model, tubes in straight configuration were used. For the 3D AVM model, tubes with different diameters were manually configured in an irregular configuration and inserted into a plastic container. Tubes of both models were completely filled with Onyx 18. For quantitative imaging analysis a standardized donut-shaped region of interest (ROI) was placed centrally around each model by the Medical Imaging Interaction Toolkit

Two different experimental in vitro models were created for this investigation: A simple but highly standardized two-dimensional (2D) tube model, and a more complex three-dimensional (3D) model, resembling an AVM [10, 12]. The 2D tube model consisted of DMSO-compatible tubes in straight configuration with a length of 30 mm, an internal diameter of 4 mm and an outer diameter of 8 mm. The 3D AVM model consisted of two different sizes of DMSO-compatible tubes, each with a length of 600 mm. A schematic description of both experimental in vitro models is illustrated in Fig. 1. As described previously, both tubes of the 3D AVM model were manually arranged in an irregular configuration, resembling an AVM, and inserted into a thin-walled, ovate plastic container with a diameter of 32 mm. Tube 1 had an internal diameter of 1.0 mm and an outer diameter of 1.8 mm, while tube 2 had an internal diameter of 1.6 mm and an outer diameter of 3.2 mm. Before filling, all models were flushed with warm saline (38.0 °C, NaCl 0.9%). Of the different available formulations of Onyx, Onyx 18 was used, as it is the most frequently used formulation and prepared in accordance with the manufacturer’s instructions. The filling was performed by manual pulsatile injection of the LEA with an average flow of 0.2 ml per minute. The tubes of both experimental models were completely filled with Onyx. The tube of the 2D tube model was filled with a total volume of 3.78 ml. Tube 1 of the 3D AVM model was filled with a total volume of 0.47 ml and tube 2 was filled with a total volume of 1.20 ml. Ten models of each type as well as ten saline filled models, serving as a control group, were investigated. Further investigation of Onyx and saline filled 2D tube models as well as 3D AVM models (*n* = 5) was performed using an experimental hemorrhage. This experimental hemorrhage with a total volume of 3 ml was placed 5.0 mm adjacent to each filled in vitro model, consisting of saline (NaCl 0.9%) mixed with contrast medium (Imeron 300, Bracco Imaging Deutschland GmbH, Konstanz, Germany) with a density similar to fresh blood (mean ± standard deviation, SD: 81.70 ± 6.25 Hounsfield Units, HU).

### Imaging and Image Postprocessing

According to clinical routine, image acquisition was performed on a multidetector, single source scanner (Somatom Definition AS, Siemens Healthineers for the experiments without the experimental hemorrhage and Somatom Definition Edge, Siemens Healthineers for the experiments with the experimental hemorrhage) with a tube voltage of 120 kV and a tube current of 20 mAs. Following the model of Daubner et al., all filled models were inserted into a custom-made CT phantom with an average density similar to brain tissue (mean ± SD: 33.19 ± 0.69 HU) [22]. For all scans the beam path was orthogonal to the model. After image acquisition the iMAR reconstruction algorithm “Neuro coils” was applied to the original images in order to generate images with metal artifact reduction. The original and post-processed CT images were reconstructed with a standard brain imaging kernel in the axial plane with a slice thickness of 4 mm.

### Quantitative Image Analysis

The Medical Imaging Interaction Toolkit (MITK; German Cancer Research Center, DKFZ, Heidelberg, Germany) was used for quantitative analysis of the imaging artifacts caused by Onyx 18 [23]. Quantitative analysis was only conducted for the images without the experimental hemorrhage. The MITK software allowed us to place a defined, donut-shaped region of interest (ROI) adjacent to and surrounding the experimental in vitro models. For quantitative analysis of the 2D tube models, 5 ROIs were set in different positions along each tube, with a distance of 4 mm (illustrated in Fig. 1). Each ROI had an internal diameter of 12 mm and an outer diameter of 52 mm. For the 3D AVM models, the donut-shaped ROIs were placed on the central image slice of the upper third, the middle third and the lower third (also illustrated in Fig. 1). The internal diameter of the ROIs was set at 36 mm while the outer diameter was set at 66 mm. Window width (w) and level/center (l) were adjusted manually to ensure an adequate placement of each ROI. To compare the degree of artifact reduction, each ROI was placed on exactly the same image slice of the original and the post-processed images. As discussed in several studies, LEA-related imaging artifacts usually consist of areas of high density and low density [8–10, 12, 17]. Therefore, we assessed the degree of imaging artifacts by calculating the SD of the HU. This method has the advantage, that artifacts in either way do not cancel out each other, as it might be the case for the mean density values.

### Qualitative Image Analysis

Regarding the degree of the LEA-related imaging artifacts, qualitative analysis of the original (no iMAR) and the post-processed images (with iMAR) in the 3D AVM model was performed by two different readers (reader 1 with 4 years and reader 2 with 8 years of experience in diagnostic imaging) on a picture archiving and communication system workstation (CENTRICITY PACS 4.0; GE Healthcare, Barrington, IL, USA). To improve the quality of the analysis, the read was reperformed after 3 months. Each reader was blinded to the type of filling (Onyx and saline) as well as to the original (no iMAR) and post-processed (with iMAR) images of the Onyx filled models. A standard brain viewing window (w:80 HU, l:40 HU) was set for analysis and readers were not allowed to adjust the window. LEA-induced artifacts were graded by a 5-point scale: (1) no artifacts; (2) mild artifacts; (3) moderate artifacts; (4) severe artifacts and (5) major artifacts.

Additional qualitative analyses by the same readers were performed for the CT scans of all filled models (2D tube models with saline and Onyx as well as 3D AVM models with saline and Onyx) with the experimental hemorrhage placed 5.0 mm adjacent to it. Therefore, a standard brain viewing window (w:80 HU, l:40 HU) was set while a manual adjustment of the window was not allowed. Definition of the experimental hemorrhage was graded by a 5-point scale: (1) severe artifacts, experimental hemorrhage not definable; (2) moderate artifacts, less than 50% of the experimental hemorrhage definable; (3) moderate artifacts, more than 50% of the experimental hemorrhage definable; (4) minor artifacts, 100% of the experimental hemorrhage definable; (5) no artifacts, 100% of the experimental hemorrhage definable. The read was reperformed after 4 weeks to improve the quality of the image analysis.

### Statistics

For statistical analysis, GraphPad Prism software (version 9.0.0, GraphPad Software, Inc. La Jolla, CA, USA) was used. Quantitative data are presented as mean ± SD. To evaluate statistical differences between the original and the post-processed CT images, the Wilcoxon matched pairs signed rank test was performed.

For qualitative analysis of the experimental in vitro models, the interreader and intrareader agreement was assessed by using the Cohen’s κ coefficient [24]. The κ values were interpreted as follows: $\leq$0.20, no agreement; 0.21–0.39, minimal agreement; 0.40–0.59, weak agreement; 0.60–0.79, moderate agreement; 0.80–0.90, strong agreement; and ≥ 0.90, almost perfect agreement [25].

To further investigate the quantitative and qualitative differences between the individual study groups and the corresponding control groups, Kruskal-Wallis test and Dunn’s test for multiple comparisons using statistical hypothesis testing were conducted. The level of statistical significance was defined as *p* < 0.05.

## Results

**Fig. 2:**
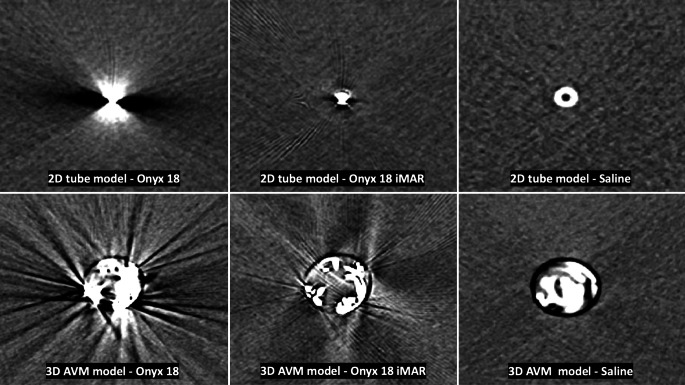
Representative CT images of the original and the post-processed images of both models and the corresponding control group. Conventional CT images are presented in a standard brain window with a width of 80 HU and a level/center of 40 HU. Comparing the same image slices of the original and the post-processed CT images, a significant reduction of the LEA-related imaging artifacts by the iterative metal artifact reduction (with iMAR) software could be observed for both in vitro models. Images of the corresponding, saline filled control groups are presented as well

**Fig. 3:**
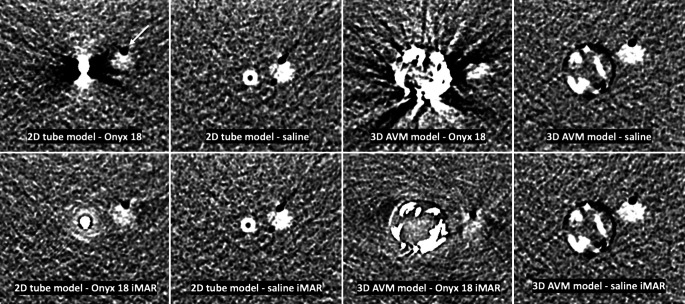
Representative CT images of the original and the post-processed images of both in vitro models with the experimental hemorrhage adjacent. CT images are presented in a standard brain window with a width of 80 HU and a level/center of 40 HU. Comparing the original (no iMAR) and the post-processed (with iMAR) images, a better definition of the experimental hemorrhage is possible after artifact reduction. Since the DMSO-compatible tubes still feature limited radiopacity, especially on the images of the 2D tube models, minor tube-associated artifacts can be observed. Note the small preparation artifact (*white arrow*) of the experimental hemorrhage

Representative CT images of the original and the post-processed recordings of both models as well as images of the related control groups are shown in Fig. 2. Original and post-processed recordings of the in vitro models with the experimental hemorrhage placed adjacent to it are demonstrated in Fig. 3.

**Fig. 4:**
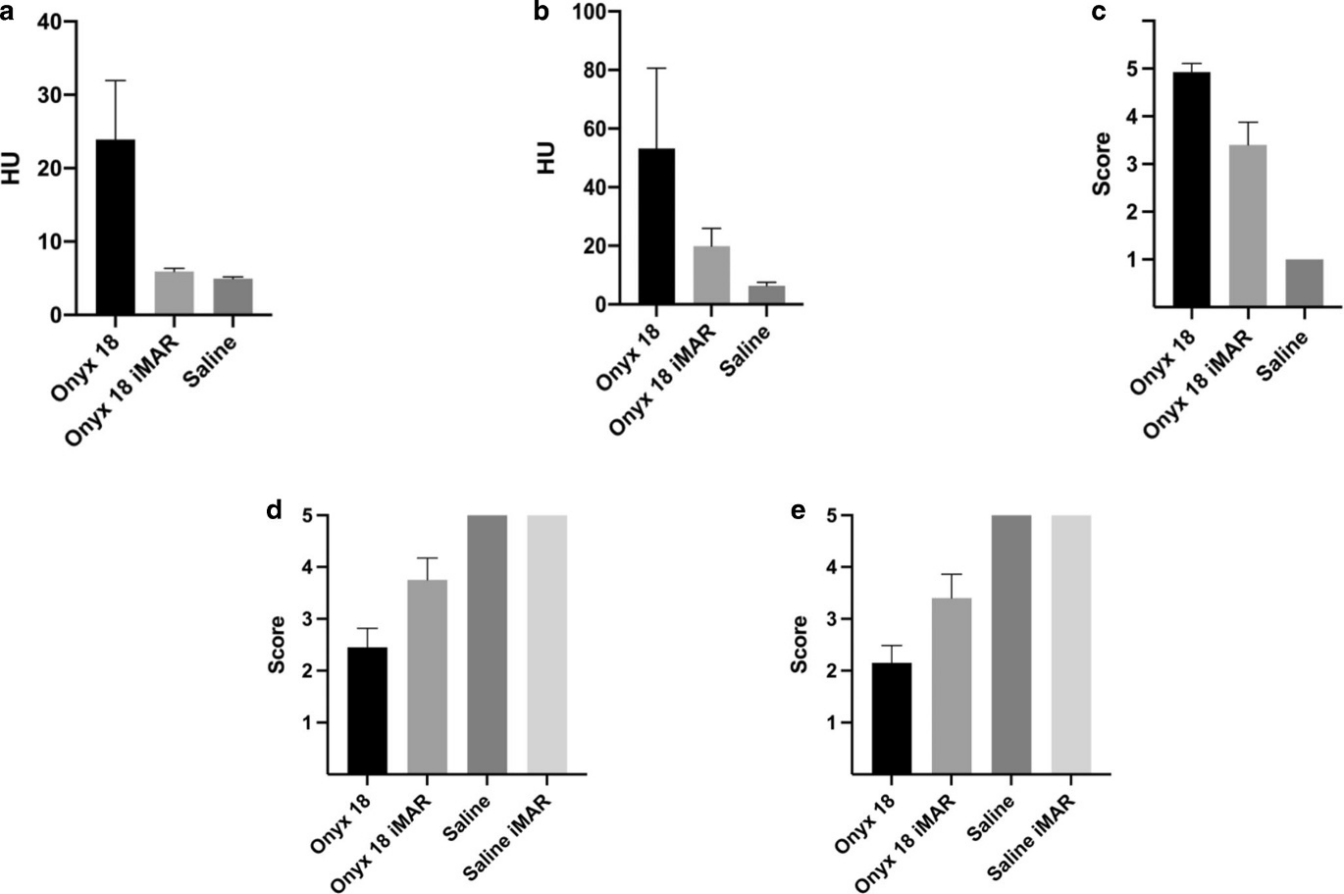
Illustration of the results of the quantitative and qualitative image analyses. The imaging artifacts caused by Onyx 18 were significantly lower in the post-processed images (with iMAR) in comparison to the original CT images (no iMAR), both in the 2D tube (**a**) and in the 3D AVM model (**b** quantitative analysis; **c** qualitative analysis by a 5-point scale). There was still a significant difference in the degree of artifacts between the post-processed images and the control group with saline-filled tubes. Qualitative analyses by a five-point scale demonstrated a better definition of the experimental hemorrhage in the post-processed images in both in vitro models (**d** 2D tube model; **e** 3D AVM model) while there was no difference in the saline filled in vitro models. Bars: mean, whiskers: standard deviation, *HU* Hounsfield units

The results of the quantitative analysis are illustrated in Fig. 4 and summarized in Table 1. Comparing the LEA-related imaging artifacts in a defined ROI of the same image slices of the original and the post-processed CT images, Wilcoxon matched pairs signed rank test showed a significantly lower degree of the artifacts in the post-processed images in both experimental models, for example, in the 2D tube model: 23.92 ± 8.02 HU (mean ± SD; no iMAR) vs. 5.93 ± 0.43 HU (with iMAR); in the 3D AVM model: 53.19 ± 53.19 HU vs. 19.91 ± 6.03 HU; *p* < 0.001).

**Table 1:** Onyx 18 related imaging artifacts before and after post-processing with the iMAR algorithm

*(A) Tubes*	*Original CT images* *(no iMAR)*	*Post-processed* *CT images (with iMAR)*	*p‑value*
Onyx 18	23.92 ± 8.02 HU	5.93 ± 0.43 HU	*p* < 0.001^a^
Saline	4.95 ± 0.22 HU	–	*p* < 0.001^b^
*(B) AVMs*	*Original CT images* *(no iMAR)*	*Post-processed* *CT images (with iMAR)*	*p‑value*
Onyx 18	53.19 ± 27.45 HU	19.91 ± 6.03 HU	*p* < 0.001^a^
Saline	6.37 ± 1.18 HU	–	*p* < 0.001^b^

The iMAR algorithm was able to reduce the Onyx 18 related imaging artifacts in conventional computed tomography in both in vitro models.

*AVM* arteriovenous malformation, *iMAR* iterative metal artifact reduction, *CT* conventional computed tomography, *HU* Hounsfield units.

^a^*p*-values of the Wilcoxon matched-pairs signed rank test to evaluate statistical differences between the original (no iMAR) and the post-processed CT images (with iMAR)

^b^*p*-values of the Kruskal-Wallis test and Dunn’s post-hoc test, respectively, to evaluate statistical differences between both study groups (2D tube models and 3D AVM models; original images (no iMAR) and with iMAR) and the corresponding control group

A summary of the results of the qualitative analysis is illustrated in Fig. 4. A detailed description of the individual findings without the experimental hemorrhage is provided in Table 2, while detailed results in consideration of the experimental hemorrhage are demonstrated in Table 3.

**Table 2:** Summary of the results of the qualitative imaging analysis in the 3D AVM models without the experimental hemorrhage

*(A)*	*Original CT images* *(no iMAR)*	*Post-processed CT images* *(with iMAR)*	*p‑value*^*a*^
Onyx 18	4.93 ± 0.18	3.40 ± 0.48	*p* < 0.001
Saline	1.00 ± 0.00	–	
*(B)*	*Onyx 18* *(no iMAR)*	*Onyx 18* *(with iMAR)*	
Saline	*p* < 0.001	*p* < 0.001	
Onyx 18 (with iMAR)	*p* < 0.001	–	

Five-point scale artifact analysis in the 3D AVM models by two different readers (Table A; shown as mean ± standard deviation)

The *P*-values post-hoc Dunn’s test for multiple comparisons using statistical hypothesis testing demonstrated further differences between the individual study groups (Table B)

*AVM* arteriovenous malformation, *iMAR* iterative metal artifact reduction, *CT* conventional computed tomography

^a^Kruskal-Wallis test

**Table 3:** Summary of the results of the qualitative imaging analyses for the definition of the experimental hemorrhage

*(A)*	*Original CT images* *(no iMAR)*	*Post-processed CT images* *(with iMAR)*	*p‑value*^*a*^
2D tube model Onyx 18	2.45 ± 0.37	3.75 ± 0.42	*p* = 0.004
3D AVM model Onyx 18	2.15 ± 0.34	3.40 ± 0.46	*p* = 0.002
2D tube/3D AVM model Saline	5.00 ± 0.00	5.00 ± 0.00	*p* > 0.999

Qualitative analyses by a five-point scale revealed a significantly better definition of the experimental hemorrhage in the post-processed (with iMAR) images in both with Onyx filled in vitro models. There was no difference between the original (no iMAR) and the post-processed (with iMAR) images for defining the experimental hemorrhage in the saline filled models

*AVM* arteriovenous malformation, *iMAR* iterative metal artifact reduction, *CT* conventional computed tomography

^a^Kruskal-Wallis test

Qualitative analysis of the 3D AVM models without the experimental hemorrhage demonstrated significantly more severe artifacts in the original images of the Onyx filled models (no iMAR), compared to the post-processed images (with iMAR) and the control group (no iMAR: 4.93 ± 0.18; with iMAR: 3.40 ± 0.48; saline: 1.00 ± 0.00). Interreader and intrareader reliability showed a strong agreement (interreader: κ = 0.886; range: 0.79–0.982/intrareader: κ = 0.817; range: 0.697–0.935) for the rating of the degree of imaging artifacts in the 3D AVM model.

Kruskal-Wallis test revealed a significant difference in the degree of artifacts between all study groups in quantitative and qualitative analysis (*p* < 0.001, respectively). Despite the distinct reduction of artifacts in the post-processed images, the Dunn’s post-hoc test showed still a significant difference in the degree of artifacts comparing the post-processed CT images (with iMAR) with the corresponding control group for both models (*p* < 0.001, respectively).

For the qualitative analyses of both experimental in vitro models with the experimental hemorrhage adjacent, the Wilcoxon matched pairs signed rank test demonstrated a better definition in the post-processed (with iMAR) images (2D tube model: no iMAR: 2.45 ± 0.37, with iMAR: 3.75 ± 0.42 [*p* = 0.004]; 3D AVM model: no iMAR: 2.15 ± 0.34, with iMAR: 3.40 ± 0.46 [*p* = 0.002]). There was no difference for definition between the original (no iMAR) and the post-processed (with iMAR) images for both saline filled models (5.00 ± 0.00, *p* > 0.999). Interreader and intrareader reliability showed a strong agreement (interreader: κ = 0.831; range: 0.727–0.935/intrareader: κ = 0.869; range: 0.776–0.962).

## Discussion

In this study, the effect of artifact reduction by the iMAR software on the imaging artifacts evoked by Onyx 18 was systematically analyzed in a 2D tube and a 3D AVM model in an experimental brain phantom. This study showed that the iMAR reconstruction algorithm is able to significantly reduce the LEA-related imaging artifacts in quantitative and qualitative image analysis in two different in vitro models.

Recently, several preclinical studies analyzed and compared the degree of imaging artifacts of different LEAs [8–10, 12]. All of these studies revealed that Onyx 18 induces the highest degree of imaging artifacts in conventional CT in comparison to less frequently used LEAs, such as Squid (Balt, Montmorency, France) or PHIL (MicroVention, Aliso Viejo, CA, USA). Further studies demonstrated that the imaging artifacts caused by Onyx 18 can have a major impact on the treatment planning and the performance of stereotactic radiotherapy of AVMs [14–18]. In consideration of these findings and the fact that the non-adhesive agent Onyx 18 is still the most commonly used LEA for embolization of intracranial vascular malformations, we decided to focus the present research on this particular embolic agent [5–7].

Several studies could demonstrate that iMAR is feasible and effective for artifact reduction in conventional CT for metallic implants, such as intracranial platinum coils or shoulder and hip protheses [20, 21]. Regarding embolic agents, there is only one scientific article available, assessing the degree of artifact reduction of Onyx 18 in aortic CT angiography after embolization [11]. So far, no study investigated software-based techniques, such as iMAR, for the reduction of imaging artifacts caused by LEAs like Onyx 18 after the embolization of vascular malformations of the brain.

The iMAR software includes different reconstruction modes which are principally based on the same algorithm, while only specific settings, like the number of iterations and the threshold for metal segmentation, are modified [26, 27]. Since our study was conducted in a brain model and the atomic number of tantalum (atomic number: 73) as relevant component of Onyx 18 (for example versus the atomic number of iodine: 53, as relevant component of the hydrophobic injectable liquid—PHIL 25; MicroVention) is very similar to the atomic number of platinum (atomic number: 78), as the main component of neurovascular coils, only the iMAR algorithm “Neuro coils” was applied [28]. In comparison to in vivo patient data, the present in vitro models as well as the brain phantom, have the advantage to feature a high level of standardization such as size and volume of the utilized tubes and the amount of LEA for filling. In contrast, the only available abovementioned study on this topic only included patients with endovascular aneurysm repair (EVAR) [11]. While for EVAR, special stent-grafts are used which have a metallic and thus an artifact generating skeleton, the tubes of the present study demonstrated only limited radiopacity [29]. Therefore, different types of DMSO-compatible tubes were tested before the preparation of the in vitro models and the tubes with the lowest radiopacity were used.

A special feature of the analysis of imaging artifacts in this study is the standardized donut-shaped ROI which was described previously [10, 12]. As an advantage over manual drawing of a ROI adjacent to an artifact producing substance, which may lead to an inaccurate and less standardized analysis of artifacts, this type of ROI takes all of the surrounding imaging artifacts into account. This type of measurement may lead to bias in comparing the degree of artifact reduction. To ensure a more accurate analysis, the ROIs were set at 3 different levels of each 3D AVM model and at 5 different levels of each 2D tube model [10, 12]. The results of this highly standardized approach could demonstrate a significant reduction of the Onyx 18 related artifacts by the iMAR algorithm in two different in vitro models in a brain phantom. Comparing both in vitro models, especially the simple 2D tube model features a high level of standardization in terms of diameter and length. While this simple configuration allows a highly standardized comparison, the more complex, 3D AVM-resembling model has the advantage to represent a human AVM in a more realistic way. Despite these advantages, there are obviously still major differences in comparison to a human AVM, especially in terms of size and morphology as well as flow dynamics. Nevertheless, the use of two different in vitro models offers the advantage of a systematic analysis with an increased informative value. Moreover, we addressed further challenges of clinical routine in our study and performed an additional analysis using an experimental hemorrhage, which outlines a further benefit of the present study. Therefore, in a first experiment, a nidus-shaped rubber latex was filled with human blood and CT scans were performed with a delay of 60 min in order to make the human hemorrhage appear hyperdense. Due to the in vitro character of the experimental brain phantom, the human hemorrhage appeared nearly isodense to the brain phantom and we therefore decided to use an experimental hemorrhage with a density similar to fresh blood, consisting of saline mixed with contrast medium. Despite this property, this composition is also the reason why in some of the post-processed images the experimental hemorrhage has lost a little of its density (see Fig. 3).

Besides the applied iMAR algorithm, there are further metal artifact reduction algorithms commercially available, for example smart MAR (GE Healthcare, Milwaukee, WI, USA), single-energy MAR (SEMAR; Canon Medical Systems Corporation, Otawara, Japan) or MAR for orthopedic implants (O-MAR; Philips Healthcare, Best, The Netherlands) [19]. So far, for none of these algorithms the possibility of reducing LEA-induced imaging artifacts has been investigated. In future studies it would be interesting to analyze the effect of these algorithms on the reduction of LEA-related imaging artifacts.

We acknowledge that this study has further limitations. The used tubes were slightly radiopaque, which might modify the degree of artifacts and thus the comparison to the corresponding control groups. Moreover, the in vitro models were not flushed continuously with saline during the LEA injection, what might have a further impact on the degree of imaging artifacts. Only one LEA was investigated and transferability to patient data is thus limited since cerebral vascular malformations are often embolized using two or more different LEAs of different radiopacity. Due to the experimental in vitro character of the brain phantom, the use of human hemorrhage was not possible and investigation was performed with an experimental hemorrhage, consisting of saline mixed with contrast medium. Moreover, according to limitations of the iMAR software license, two different CT scanners were used for image acquisition.

## Conclusion

The iMAR algorithm can significantly reduce the imaging artifacts evoked by Onyx 18 in CT in two different in vitro models in a brain phantom. Applying iMAR could thus improve the accuracy of postprocedural CT imaging after embolization with Onyx in clinical practice.
